# Welcome to a new era for *Clinical and Molecular Allergy*

**DOI:** 10.1186/s12948-015-0014-z

**Published:** 2015-04-15

**Authors:** Enrico Maggi

**Affiliations:** Allergology and Clinical Immunology Department of Clinical and Experimental Medicine, University of Firenze, Largo Brambilla 3, 50134 Firenze, Italy

It is my pleasure and honour to introduce the new *Clinical and Molecular Allergy(CMA)*, an open access, peer-reviewed journal published by BioMed Central, which in 2015 has re-launched as the official journal of the Italian Society of Allergology, Asthma and Clinical Immunology (SIAAIC). SIAAIC is the oldest scientific society of the discipline in Italy which is strongly represented on the national territory with more than 650 active members working in University structures, Hospital departments and Territorial outpatients' clinics. SIAAIC is the only Italian society represented at the European level (EAACI).

The editorial team of the journal has been completely renewed, and can be accessed here(http://www.clinicalmolecularallergy.com/about/edboard). The Editor-in-Chief is the president-elect of SIAAIC and the new Deputy and Section Editors are members of the National Committee of the Society, all displaying an international relevance in the field. The new members of the Editorial Board, competent to cover the majority of fields of basic, translational and clinical Allergology and Clinical Immunology, are strongly motivated scientists, many of them known worldwide. They are responsible for the scientific quality and originality of publications in addition to serving as journal advocates. The primary objective of the editorial team is to reach and maintain a strong Impact Factor within few years and the main aims of the journal are:

I. To offer a prompt publication of results from original research on the up-to-date topics of experimental and clinical allergy and immunology for scientists of our discipline.
II. To share opinions among the readers with top scientists and clinical practicioners.
III. To have a very easy instrument for students and young specialists to update novelties in semestral selected reviews focused on the section topics.
IV. To share knowledge on fields of common interest with other physicians and researchers interested in allergy and clinical immunology.

Six editorial sections have been chosen to cover the majority of topics in basic translational and clinical allergy and immunology. Sections include asthma, food, drug and insect allergy, clinical and molecular allergy, allergy immunotherapy, biologicals and clinical immunology. Articles may range from pathogenetic studies on diseases to clinical trials on novel therapies, giving insights into underlying mechanisms of diseases, new methodologies and other traslational and clinical topics which inform the specialist and contribute to improve the diagnosis and management of patients.

SIAAIC have chosen to affiliate with *CMA* for its open access publishing model [1-4]. Open access journals have the potential to reach a much larger set of readers than any subscription-based journal, in print and online [5]. Studies have suggested a correlation between open access, higher downloads and higher citations, leading to a higher Impact Factor [6]. Furthermore articles are freely and universally accessible online, so an author's work is available to readers at no cost and not limited by their library’s budget. This ensures that author’s work is disseminated to the widest possible audience. Additional advantages include:

I. Authors hold copyright for their work and grant anyone the right to reproduce and disseminate the article.
II. The journal’s articles will be archived in PubMed Central [7]. This complies with the policies of a number of funding bodies as the Wellcome Trust, NIH and Howard Hughes Medical Institute [8,9].
III. A country's economy will not influence its researchers' ability to access articles because resource-poor countries will be able to read the same material as wealthier ones [10].

SIAAIC wants to make this journal available to young scientists (from the society, but not only limited to) who need to quickly publish their results and receive feedback from experienced colleagues to proceed with their studies. The availability of semestral reviews on very hot topics from high level scientists, one on clinical immunology and the other in Allergology, as well as annual editorials highlighting the most relevant improvement on their fields from the Deputy and Section editors may contribute to increase contacts with the *CMA* website from a large interested audience. This will further develop the journal and its Impact Factor.

Allergology and Clinical Immunology is a quickly evolving discipline where novelties must circulate in real time. An open access journal as *CMA* is needed in this field since is the most easy platform to communicate new data and to share opinions or criticisms among scientists, specialists and clinicians of close disciplines. The rapid dissemination of the new knowledge guaranteed by the journal will greatly enhance the value of research in the field.

In the era of several exciting discoveries on basic immunology and pathophysiological mechanisms of immuno-mediated diseases, of new diagnostic laboratory technologies for such disorders and novel therapeutical drugs as biological/biosimilar agents, the journal plans to publish periodic reviews on very hot topics from high level scientists as well as one annual editorial highlighting the most relevant improvement on the fields. The open access platform of *CMA* to research on basic and clinical allergy and immunology eliminates obstacles and challenges to dissemination of such knowledge and provides a platform for rapid discovery.

As a standard CMA will publish:

I. **Research** articles reporting original data from clinical and basic research providing insights also into physiopathological mechanisms, novel methodologies and therapeutical protocols.
II. **Reviews** giving exhaustive and authoritative overviews of any subjects within the journal’s scope, including clinical molecular aetiopathogenetic aspects of allergy and immuno-mediated disorders.
III. **Case Reports** providing molecular insights and mechanisms governing common and rare diseases.
IV. **Commentaries** as short focused articles reporting opinions on different issues within the journal subjects.
V. **Correspondences** in the form of a re-analysis of a previously published articles, or a reply to this re-analysis from the authors of the original publication, or a short article that may not cover “standard research” but is relevant for the specialists.

Of note, all articles will be published online immediately upon acceptance (after peer review) and soon after listed in PubMed.

*CMA* operates a closed peer review process. Submissions felt to be suitable for consideration will be sent for peer review with appropriate independent experts. Handling Editors will make a decision based on the reviewers’ reports and authors are sent these reports along with the editorial decision on their manuscript. Reviewers have to evaluate originality and novelties of papers, taking into account potential plagiarism, subject’s privacy and ethical aspects. The journal operates a closed, single blined peer review process whereby reviewers will be treated anonymously. The *CMA* Editors agreed to adhere the Code of conduct for Editors-in-Chief and the publisher’s policies. They check manuscripts on submission to ensure they conform with these policies.

I hope that the new *CMA* may become a global forum for the exchange of research and information on allergy, asthma and clinical immunology, an exciting opportunity to publish original results for young specialists and scientists.

Enrico Maggi, MD

Editor in Chief, *Clinical and Molecular Allergy*
